# Supplementary material for: Disentangling neocortical alpha/beta and hippocampal theta/gamma oscillations in human episodic memory formation
Source: Neuroimage. 2021 Nov 15;242:118454. doi: 10.1016/j.neuroimage.2021.118454 (PMC8463840; doi:10.1016/j.neuroimage.2021.118454)

**Supplementary Figure 2.** Time-frequency plot of corrected, source-reconstructed, hippocampal spectral power (averaged across all trials and source-reconstructed hippocampal voxels; z-transformed using the mean and standard deviation of the spectral power 500ms prior to stimulus onset) during sequence presentation and mnemonic binding. Gamma power shows a small increase during mnemonic binding. Note that such increases may be masked by averaging: as gamma power fluctuates as a function of theta phase, and theta phase is not necessarily aligned across trials, averaging across trials may underestimate the magnitude of gamma power here.


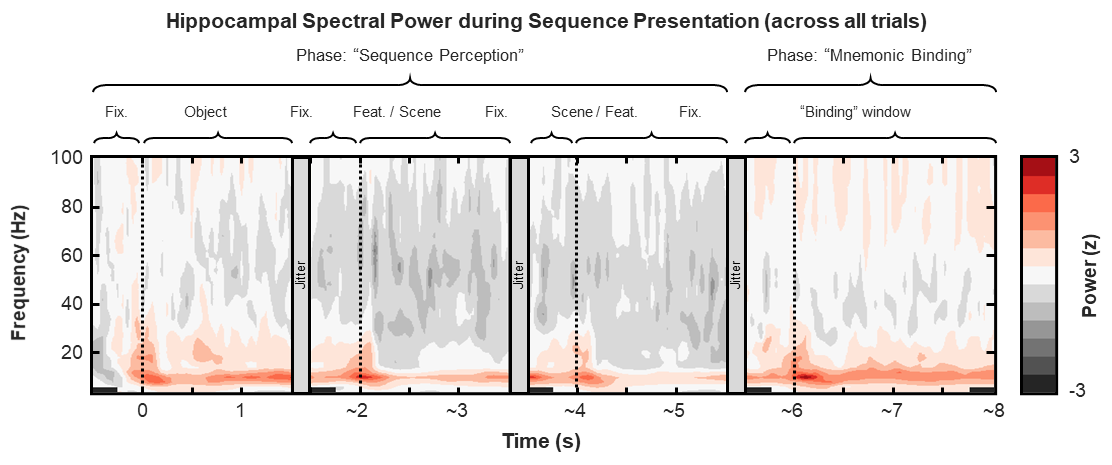


**Frequency (Hz)**

**Power (a. u.)**

**Theta Spectral Peaks**

**(a)**

**(b)**

**Frequency (Hz)**

**Power (a. u.)**

**Gamma Spectral Peaks**

**Supplementary Figure 1.** Spectral peaks in the hippocampus during mnemonic binding. **(a)** Theta spectral peaks (red line: spectral power; dotted grey line: linear fit; black line: detected peak). Note that in some instances (sub-02, sub-08 and sub-09), the peak was embedded in the cortical alpha peak. Therefore, the identified peaks were cross-checked by taking the first derivative of the plotted signal and confirming that the derivative peak overlapped with the originally detected peak. **(b)** Gamma spectral peaks (red line: spectral power; dotted grey line: linear fit; black line: detected peak).


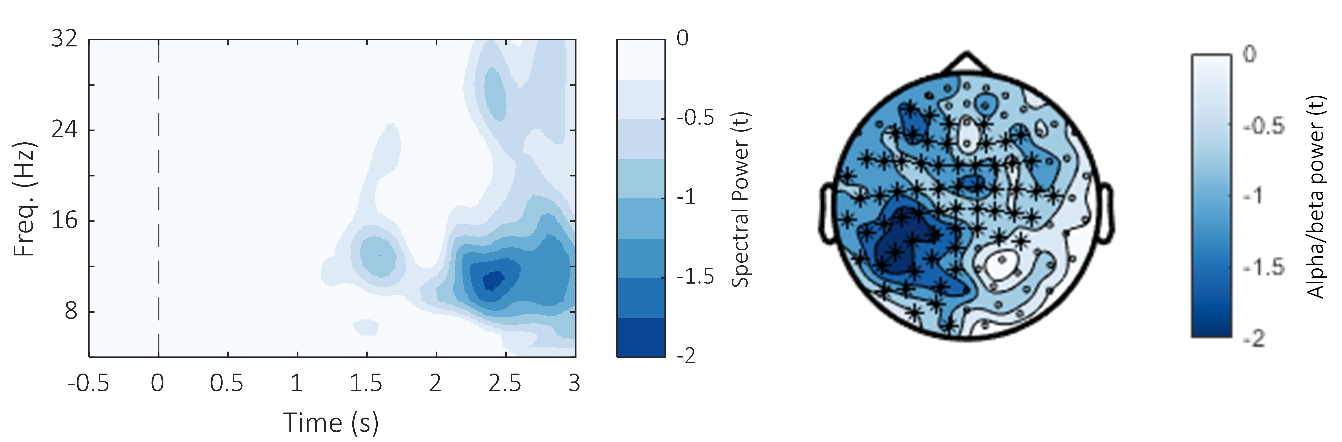


**Supplementary Figure 3.** In the main text, it is proposed that alpha/beta power decreases scale with the amount of information present in the cortex – a hypothesis that can also be generalised to the retrieval data (i.e., decreases in alpha/beta power during memory retrieval correlate with the number of items recalled). Indeed, when testing this hypothesis, we found that alpha/beta power decreases during retrieval correlated with the number of items recalled (p_corr_ = 0.027, Cohen’s d_z_ = 0.59, cluster size = 1311, mean t-statistic within cluster = -2.42). This supports our current hypothesis and conceptually replicates findings from similar experiments that have focused alpha/beta power during memory retrieval (Karlsson et al., 2020; Martín-Buro et al., 2020).

No memory-related change in hippocampal theta-gamma coupling was observed during memory retrieval (p = 0.161).

**Supplementary Figure 4.** Stimulus-specific memory effects. During the presentation of both feature (top) and scene (bottom) stimuli, the magnitude of the alpha/beta power decrease over occipital regions predicted later recall success [feature: p_clus_ = 0.017; scene: p_clus_ = 0.032]. The time-frequency plots (left) consist of data averaged across the channels included in the topographic cluster. The topographic plots (middle) consist of data averaged across times/frequencies included in the significant cluster [feature: time range 400-1000ms; frequency 8-15Hz; scene: time range 600-1200ms; frequency 8-18Hz]. The source plots (right) were generated using the times/frequencies included in the significant cluster (matching those used for the topographic plots). Notably, when correlating the resulting t-statistics (as visualised here) across time, space and frequency, we observed a significant spatio-spectro-temporal similarity between the two memory effects (r = 0.135, p < 0.001), indicating that these effects have similar neural origins.

No memory-related change in hippocampal theta-gamma coupling was observed during memory retrieval (p = 0.161).


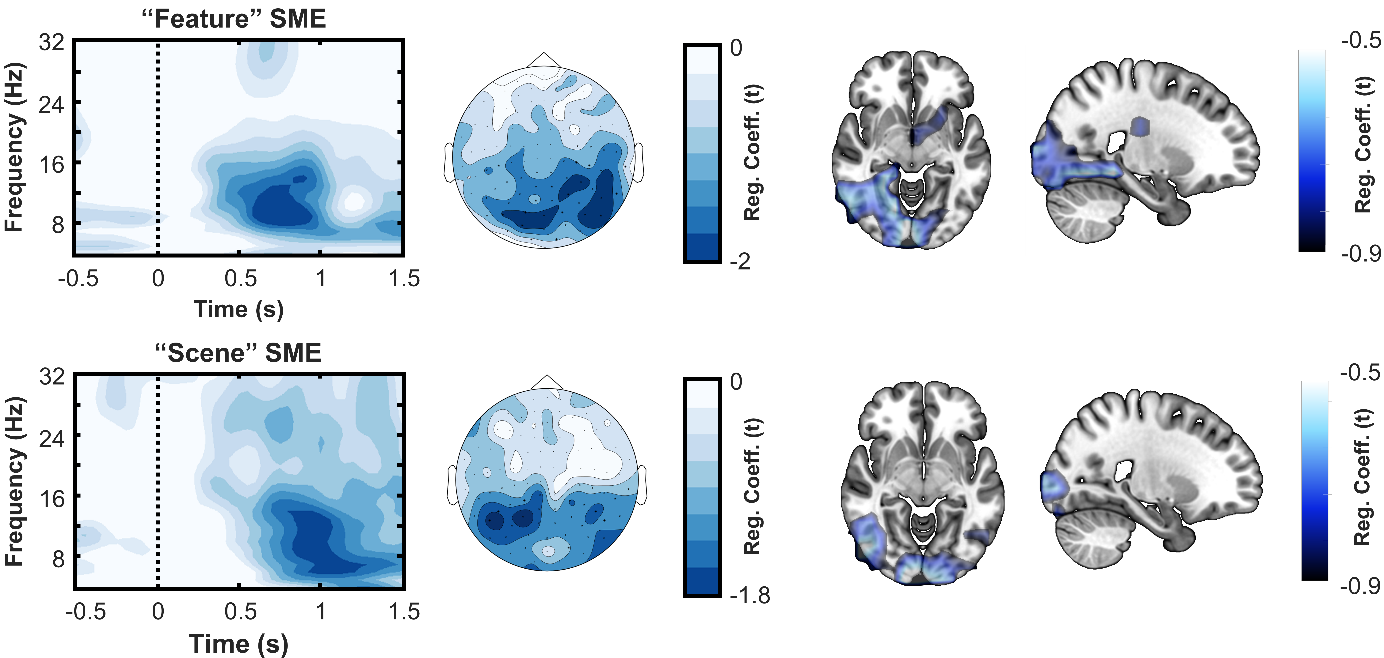


**Supplementary Figure 5.** Hippocampal theta-gamma phase-amplitude coupling during sequence perception, as a function of presentation position. Phase-amplitude coupling did not consistently increase as a function of presentation position [one-tailed t(16) = 1.34, p = 0.099; see boxplot / scatterplot on left for the model fits of individual participants]. While there seemed to be a descriptive, monotonic increase in coupling as the sequence progressed across participants (middle plot), inspection of individual participant trends (right plot) suggests this monotonic increase is not a consistent phenomenon across participants.


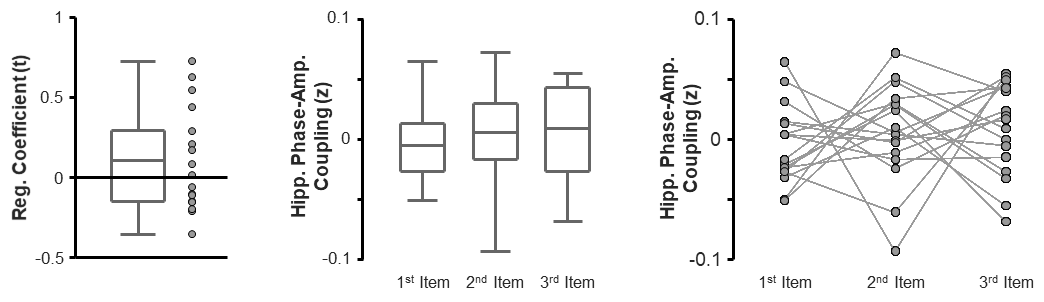


**Supplementary Figure 6.** Histogram visualising the distribution of t-statistics for theta-gamma phase-amplitude coupling for every searchlight of the source-reconstructed brain. The statistic for the hippocampal region-of-interest (ROI; solid line) was substantial greater than the majority of other searchlight-based regions-of-interest that matched the size of the hippocampus. No memory-related change in hippocampal theta-gamma coupling was observed during memory retrieval (p = 0.161).


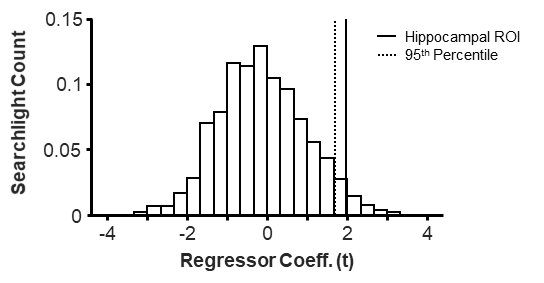


**Supplementary Figure 7.** Neocortical alpha/beta power decreases during perception correlate with increases in hippocampal theta-gamma coupling during mnemonic binding on a trial-by-trial level. No effect was observed when correlating these metrics at other epoch combinations (e.g., correlating alpha/beta power during binding with theta/gamma coupling during binding).

For each participant, a linear model [consisting of (1) a constant, (2) neocortical alpha/beta power during perception (in the cluster described in the main text), and (3) number of items recalled] was used to predict hippocampal theta-gamma coupling during mnemonic binding. The second co-efficient (i.e., alpha/beta power) for each participant model was then standardised and pooled for a one-tailed t-test (assuming a negative relationship between neocortical alpha/beta power and hippocampal theta-gamma coupling). Indeed, we found that the more that alpha/beta power decreased during sequence perception, the more hippocampal theta-gamma coupling increased during the following binding window [t(16) = -1.88, p = 0.038]. Note that this cannot be attributed to a phantom correlation induced by both MEG metrics correlating with the number of items recalled, as this source of variance was accounted for in the participant-specific linear models. No similar effect was observed for any other combination of power*PAC correlations.


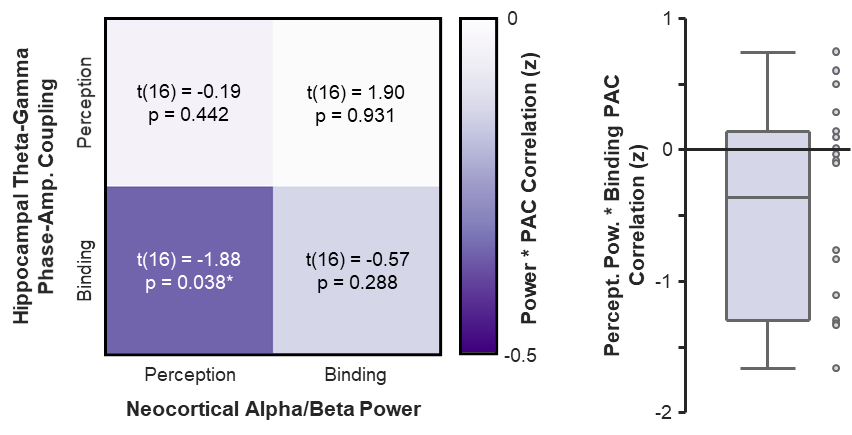

Supplement: Supplementary file 1 [file mmc1.docx]
